# Long-Term Effects of Botulinum Toxin Complex Type A Injection on Mechano- and Metabo-Sensitive Afferent Fibers Originating from *Gastrocnemius* Muscle

**DOI:** 10.1371/journal.pone.0140439

**Published:** 2015-10-20

**Authors:** Guillaume Caron, Tanguy Marqueste, Patrick Decherchi

**Affiliations:** Aix-Marseille Université (AMU) and Centre National de la Recherche Scientifique (CNRS), UMR 7287, Institut des Sciences du Mouvement: Etienne-Jules MAREY (ISM-EJM), Equipe, Plasticité des Systèmes Nerveux et Musculaire, Parc Scientifique et Technologique de Luminy, Faculté des Sciences du Sport de Marseille, CC910 - 163 Avenue de Luminy, F-13288, Marseille, cedex 09, France; Institute Pasteur, FRANCE

## Abstract

The aim of the present study was to investigate long term effects of motor denervation by botulinum toxin complex type A (BoNT/A) from *Clostridium Botulinum*, on the afferent fibers originating from the *gastrocnemius* muscle of rats. Animals were divided in 2 experimental groups: 1) untreated animals acting as control and 2) treated animals in which the toxin was injected in the left muscle, the latter being itself divided into 3 subgroups according to their locomotor recovery with the help of a test based on footprint measurements of walking rats: i) no recovery (B0), ii) 50% recovery (B50) and iii) full recovery (B100). Then, muscle properties, metabosensitive afferent fiber responses to potassium chloride (KCl) and lactic acid injections and Electrically-Induced Fatigue (EIF), and mechanosensitive responses to tendon vibrations were measured. At the end of the experiment, rats were killed and the toxin injected muscles were weighted. After toxin injection, we observed a complete paralysis associated to a loss of force to muscle stimulation and a significant muscle atrophy, and a return to baseline when the animals recover. The response to fatigue was only decreased in the B0 group. The responses to KCl injections were only altered in the B100 groups while responses to lactic acid were altered in the 3 injected groups. Finally, our results indicated that neurotoxin altered the biphasic pattern of response of the mechanosensitive fiber to tendon vibrations in the B0 and B50 groups. These results indicated that neurotoxin injection induces muscle afferent activity alterations that persist and even worsen when the muscle has recovered his motor activity.

## Introduction

*Botulinum* toxin complex type A (BoNT/A) is currently used to treat numerous medical conditions such as dystonia, neuromuscular disorders or pain. Its effects start between 2 and 5 days after injections and are maintained between 3 and 6 months [1]. BoNT/A exerts its action by preventing the exocytosis of acetylcholine vesicles at the neuromuscular junction eliciting flaccid paralysis [2,3]. Furthermore, BoNT/A induces central alterations [4–6] such as inhibition of glutamate [7], substance P [8,9], calcitonin gene related peptide (CGRP) [9,10] and to a lesser extend gamma-aminobutyric acid (GABA) release [11]. Those central changes could be due to indirect action of the toxin (decrease of the postsynaptic element activation following the decrease in presynaptic element activation) or to the toxin retrograde transport to the spinal cord (SC) and transcytosis [12–14].

BoNT/A also prevents acetylcholine releases by γ-motor endings in intrafusal muscle fibers [15,16]. The lack of γ-motor endings discharge in the injected muscle induce an intrafusal muscle fiber relaxation and then a decrease of afferent (Ia and II) inputs originating from spindles. At the spinal level, these changes reduce the direct excitation of agonist motoneurons and the indirect inhibition of antagonist motoneurons leading to a larger relaxation of agonist muscle (BoNT/A treated)[17]. Thus, the BoNT/A alters the central adjustments by mechanosensitive (Ia and II from muscle spindle and Ib from Golgi tendon organ)[18] and metabosensitive (III and IV) muscle afferent fibers [19]. Ia afferents detect muscle length and velocity while II afferents are mainly sensitive to instantaneous changes in muscle length [18,20]. Ib afferents are sensitive to forces variations [21–23]. Muscle afferent fibers from groups III and IV detect change in muscle metabolism [24,25] and in intramuscular pressure [26]. They are selectively stimulated during and after muscle fatigue [27] or by different agents such as bradykinin, capsaicin [28], lactic acid, H^+^ [25,29], arachidonic acid, prostaglandin [25], thromboxane A2 [30] and potassium chloride [31,32].

In a recent paper, we show that BoNT/A induces alterations in mechano- and metabosensitive afferent fibers when the muscle is to its paralysis apogee [19]. However, data are missing when the toxin is degraded and during muscle recovery.

The main purpose of the present study was to measure, over recovery time, the effects of *gastrocnemius* BoNT/A injection on afferent fibers involved in the sensorimotor loop. The muscle afferent discharges from groups III and IV were recorded after direct electrical muscle stimulation inducing fatigue and intra-arterial injections of potassium chloride or lactic acid while the mechanosentive afferents discharges were recorded after tendon vibrations. The muscle properties (muscle weight, tetanus threshold, twitch amplitude and Fatigue Index) were also measured.

## Materials and Methods

### 1. Animals

Twenty seven adult male Sprague Dawley rats, weighting 300–400g (Centre d’Elevage Roger Janvier^®^, Le Genest Saint Isle, France), were housed in smooth-bottomed plastic cages at 22°C with a 12-h light/dark cycle. Food (Safe^®^, Augy, France) and water were available *ad libitum*. An acclimation period of 1 week was allowed before the initiation of the experiment. Animals were randomized in 2 experimental groups: 1) untreated animals acting as control (Control, n = 6) and 2) treated animals in which the toxin was injected in the left muscle, the latter being itself divided into 3 subgroups according to their locomotor recovery with the help of a test based on footprint measurements of walking rats: i) no recovery (B0, n = 9, twelve days post-injection), ii) 50% recovery (B50, n = 7) and iii) full recovery (B100, n = 5).

### 2. Ethical approval

Anesthesia and surgery were performed according to the French law on animal care guidelines. The Animal Care Committees of *Aix-Marseille Université* (AMU) and *Centre National de la Recherche Scientifique* (CNRS) approved our protocols. Individual conducting researches were listed in the authorized personnel section of the animal research protocol or added to a previously approved protocol (license n°A 13.013.06). Furthermore, experiments were performed following the recommendations provided in the *Guide for Care and Use of Laboratory Animals* (U.S. Department of Health and Human Services, National Institutes of Health) and in accordance with the European Community’s council directive of 24 November 1986 (86/609/ EEC).

Animals did not present clinical sign of pain or unpleasant sensation (i.e. screech, prostration, hyperactivity, anorexia) and no paw-eating behavior were observed through the study.

### 3. Toxin injection procedure

Animals from B0, B50 and B100 groups were anesthetized by an intra-muscular injection of solution containing a ternary mixture [5 ml of ketamine, (100 mg/kg, Virbac^®^, Carros, France); 2.5 ml of largactyl (1.2 mg/kg, Avensis^®^, Paris France); 2 ml of domitor (20 mg/kg, Novartis^®^, Mississauga, Canada); 0.1 ml/100 g of body weight, IM]. Lyophilized botulinum toxin complex type A (Dysport 500^®^, Beaufour Ipsen Pharma, Boulogne-Billancourt, France) was extemporary reconstituted in normal saline solution to obtain a 15 U.ml^-1^ solution. BoNT/A solution was injected in the inferior and superior parts of the two left *gastrocnemius* muscle heads (i.e., 4 injections per rat = 2 injections in the lateral and 2 injections medial muscle heads). Each animal received a total of 15 U.kg^-1^ in order to ensure a full neurotransmitter release blockage [33–35]. As previously described, effects of this toxin start between 2 and 5 days after injection and are maintained between 3 and 6 months [1]. Furthermore, it was also described that functional loss was still maximal at the twelfth day [19].

### 4. Functional assessment of hind limb loss

All the animals were familiarized to go through a walking track apparatus (150 cm long, 9 cm wide and 40 cm high) that was used to perform the functional tests during the pre-surgical week. Briefly, all the animals were conditioned to walk homogeneously into the recording apparatus three times per day and five days during the week before surgery. Heavy lighting was provided with two 500 W spots and a dark box was positioned at the chamber’s end to promote walking. The Sciatic Functional Index (SFI) was calculated with the formula [-38.3(ePL-nPL)/nPL+109.5(eTS-nTS)/nTS+13.3(eIT-nIT)/nIT-8.8] adapted by Varejao et al., [36] from the Peroneal Functional Index [37–39] to evaluate the functional integrity of the sciatic nerve based on footprint measurements of walking rats. Footprint's length (PL, or longitudinal distance between the tip of the longest toe and the heel), total toes spreading (TS, or cross-sectional distance between the first and fifth toes) and intermediate toe spread (IT, distance between the second and fourth toes) are the main factors altered by blocking release of acetylcholine at the neuromuscular junction due to motor loss of the toe flexor, foot plantaflexor and everters. In order to do so, the animal hind feet were dipped into Chinese ink (Swop-Pads^®^, Trodat, France) and the footprints were recorded on paper track and manually analysed. The normal/uninjected (n, right) footprints and the contralateral experimental/injected (e, left) footprints were compared. The loss rate of the SFI was defined on a score of -100 to 0, where 0 (perfect symmetry) to -20 represents a normal function and -100 a total failure. Footprints were obtained on a daily, weekly or monthly basis and analysed from the injection day (before injection) to the day when the wished functional recovery was obtained (no recovery, 50% recovery and full recovery).

### 5. Electrophysiological recordings

At the end of the functional assessment period, rats were anesthetized with urethane (1.1 g.kg^-1^ i.p.), and atropine (1 mg.kg^-1^, i.p.) was administered to reduce airway secretions. Animals were tracheotomized and artificially ventilated (Harvard^®^ volumetric pump: rate 40–60 min^-1^, tidal volume 2–4 ml; Southmatick, MA USA). Animal temperature was maintained between 36–37°C with a blanket controlled by rectal temperature probe. A polyethylene catheter was inserted into the right femoral artery from the non-injected hindlimb and pushed up to the fork of the abdominal aorta in order to transport supplemental dose of anaesthetic and chemicals (i.e., potassium chloride [KCl] and lactic acid [LA]) to the controlateral muscle. This catheter was positioned in order to let the blood flow freely to the left lower limb muscles. Animals were positioned in ventral *decubitus*. Dissection at the middle thigh level was carried out to expose the right common sciatic nerve. With microsurgical techniques and an operating microscope (x40, MZ75^®^, Leica, Heerbrugg, Switzerland), a longitudinal incision was made along the lateral thigh and upper leg. Then, the tibial nerve, a branch of the sciatic nerve, was dissected free from surrounding tissues over a 20 mm length and immersed in paraffin oil to avoid dehydratation. Two tungsten stimulating electrodes (inter-electrode distance: 4–5 mm) were placed to the surface of the *gastronemius* muscle. The nerve was cut and pair of cuff electrodes was placed on the proximal tibial nerve end for stimulation or recordings. The ankle and the knee were firmly held by clamps on a horizontal support in order to avoid disturbing movements and to maintain the 90° knee and ankle joint angle during electrical nerve stimulations.

#### Twitch contraction and tetanus threshold measurements

The contractile response of the *gastrocnemius* to nerve or muscle stimulation (twitch contraction, which is a reflection of the tension generated in the muscle) was obtained with a neurostimulator (Grass S88K^®^, Grass Technologies, Natus Neurology Inc., Warwick, Rhode Island, USA) delivering single rectangular pulses (duration: 0.1 ms, frequency 0.5 Hz) through an isolation unit and measured with an isometric strain gauge (Micromanometer 7001^®^, Ugo Basile SRL, Comerio VA, Italy) fixed to the distal part of the *gastrocnemius* tendon (calcaneum tendon). The intensity of the stimulation was increased until the maximal response was found. Twitch contraction was recorded with Biopac MP150^®^ system (sampled at 2000 Hz, filtered with Low Pass at 150 Hz) and analyzed (AcqKnowledge^®^ 3.7.3 software) in terms of peak amplitude (A in Newton, N).

Then, the tetanus threshold, defined as the frequency from which we observed a sustained contraction with no relaxation between twitches, was recorded. After determining a threshold able to elicit a twitch, pulse train intensity was set to a supramaximal level. Tetanic threshold was obtained by increasing frequency by 5 Hz steps. The voltage was 20% higher than the voltage evoking a maximal twitch. The duration of stimulus trains was 500 ms, and trains were repeated each second to produce a series of contractions. Pulse duration was 2 ms and five single stimulations were delivered in each 500 ms train (10 Hz).

#### Electrically-induced fatigue

Half an hour of rest after the last stimulations used to evoke twitch and tetanus contractions, a 3-min electrically-induced muscle fatigue (EIF) was performed. For this purpose, rhythmic muscle contractions were produced by the neurostimulator (Grass S88K^®^) delivering rectangular pulse trains to the pair of electrodes placed on the muscle surface (pulse duration: 0.1 ms; frequency: 10 Hz, i.e., 5 shocks in each 500 ms train; duty cycle: 500/1000 ms, voltage range: 5 to 8 volts). The voltage was supramaximal, i.e., +20% higher than that used to elicit a maximal twitch contraction. Muscle strength was recorded from the beginning to the end of muscle electrical stimulation with the isometric strain gauge (Micromanometer 7001^®^) and fatigue was assessed from the decay of force throughout the 3-min EIF period. Thus, the fatigue index (FI), defined conventionally as the percentage of the force lost at the end of the 3-min EIF trial, was calculated [40,41].

#### Metabosensitive afferent recordings

Furthermore, during repetitive muscle stimulation, the response of muscle afferent fibers from groups III and IV was also recorded (Biopac MP150^®^ and AcqKnowledge^®^ software). The neural signals were amplified (10 to 100 K) and filtered (filtered with Low Pass at 150 Hz) with a differential amplifier (P2MP^®^ SARL, Marseille, France) and referred to a ground electrode implanted in a nearby muscle. Signals were fed into pulse window discriminators (P2MP^®^ SARL, Marseille, France) which simultaneously analyzed afferent spikes. The output of these discriminators provided noise-free tracings (discriminated units) which were computed using data analysis system (Biopac AcqKnowledge^®^ software). Before applying stimulus known to activate the afferent fibers from group III and IV, a baseline recording (F_impluses_.s^-1^) was achieved to ensure that the discharge rate remained stable. The recording was considered suitable only if the fluctuation of baseline impulse activity ranged between 100–103%. Consequently, changes in firing rate were related only to the stimuli applied and not to environmental conditions. The discharge rate was averaged over a 1-min period preceding EIF (baseline activity), and its change was measured during the first minute period following stimulation. Thus, changes were expressed in percentage of the corresponding baseline discharge rate; i.e. baseline discharges corresponded to 100%.

After a period of rest of one hour, KCl (0.5 ml; 1, 5, 10, and 20 mM) and LA (0.1 ml; 0.5, 1, 2, and 3 mM) were randomly injected into the contralateral artery while nerve discharge was continuously recorded. The injections, which required 5–10 s to be completed, were washed with 0.1 ml of normal saline. Baseline afferent activity was averaged on 1 min long period pre- and post-injection. The post-stimulus discharge firing rate was compared to the corresponding baseline discharge rate and variations were expressed in percentage of the corresponding baseline discharge firing rate. There was a 10 min delay between each injection in order to let the afferent activity go back to its baseline activity. Thus, like in a previous study [19,27] the dose/response curves to KCl and LA from femoral nerve were drawn.

#### Mechanosensitive afferent recordings

Muscle mechanosensitive fibers are activated by tendon vibration without activating group muscle metabosensitive afferents [42] in a range of 10–100 Hz [27,43], depending on the animal species and also on application through the skin or directly on the muscle tendon. Static spindle afferents (type II) and also Golgi tendons organs (type Ib, 50% are discharging with no volunteer contraction) are activated by low-frequency vibrations, whereas dynamic spindle afferents (type Ia) are activated by high-frequency vibrations [44]. Rectangular shocks were delivered perpendicularly to the longitudinal muscle axis on calcaneal tendon by a mechanical vibrator (Ling Dynamic System^®^, LDS group, Herfordshire, U.K) connected to a frequency generator (GenTrad Function Generator GF763AF, ELC^®^, Annecy, France). Vibrations were applied for 5-sec periods and frequency was increased step-by-step from 10 to 100 Hz, with 5-sec rest between each step, while the discharge of afferent units was recorded. The maximal mechanosensitive afferents discharge rate elicited by tendon vibrations was considered as the reference discharge rate (100%). The discharge rate induced by the others frequencies of vibrations were expressed in percentage of the corresponding reference discharge rate.

### 6. Muscle atrophy

At the end of the electrophysiological recordings, all rats were killed by a 3 ml intra-arterial overdose of pentobarbital sodium solution (Pentobarbital Sodique^®^, 0.6 g.kg^−1^ Sanofi Santé Animal, Sanofi France, Paris France). Left *gastrocnemius* muscles were harvested and immediately weighted on a precision scale (Navigator™ N30330 model, OHAUS Corp., Parsippany, NJ, USA). Muscle mass was measured using a muscle weight/body weight ratio and atrophy was evaluated by comparing to the Control group ratio.

### 7. Statistics

Data processing was performed using a software program (SigmaStat^®^ 2.03, Statistical software, San Jose, CA, USA). Data were expressed as mean ± Standard Error of the Mean (SEM).

For functional assessments, data concerning each animal were individually identified, in order to allow follow-up over time. Differences were tested by two-way repeated analysis of variance (ANOVA test, factors: group x timing) completed by a Student-Newman-Keuls *post-hoc* test to compare the SFI score, the effect of EIF and drug injections on metabosensitive afferent discharges, mechanosensitive afferent discharges to tendon vibrations and the muscle properties in the different groups. Finally, the intergroup comparison of the percentage of mechanosensitive fibers responding to mechanical vibration was examined with a Chi square test (χ^2^).

Results were considered statistically significant if the p-value fell below 0.05.

## Results

### 1. Functional assessment of hindlimb locomotor property

In the B0 group, measurement of the SFI indicated that functional loss started the first day post-injection, reached a minimal value the third day and remained at this minimum until the twelfth day (when electrophysiological recordings were performed). As previously shown [19], this functional score was significantly lower (p<0.001) than those recorded before injection or in Control group. No difference was observed with the B50 and B100 groups. In the B50 and B100, a functional recovery was observed at the 60^th^ day post-injection. In the B50 group, animals reached a mean score of -47.47±0.57 after 128.43±7.43 days. In the B100 group, animals reached a mean score of -18.78±3.23 after 371.83±24.82 days.

### 2. Muscle properties

Intergroup comparison indicated that there is a significant differences (p<0.001) in twitch amplitude, tetanus threshold, fatigue index (FI), muscle weight and muscle weight/body weight ratio.

In the B0 (twelve days after the injection), the electrical stimulation applied on tibial nerve did not elicit a muscle contraction (in a voltage range known to induce one). However, muscle stimulation induced a twitch with an amplitude significantly lower (p<0.001) than that of Control group. The tetanus threshold was also lower (p<0.05) than that of Control group. The FI obtained during the 3-min EIF (10 Hz) was not different than the Control group but significantly higher than the FI obtained in the B50 (p<0.001) and B100 (p<0.01) groups. Finally, the left muscle weight and the muscle weight/body weight ratio were significantly lower (p<0.001) than the Control group, indicating a muscle atrophy.

In the B50 group, muscle stimulation elicited a muscle contraction with a lower amplitude (p<0.01) than that of the Control group. The FI was significantly lower than that of the Control (p<0.01) and B0 (p<0.001) groups. Finally, the left muscle weight and the muscle weight/body weight ratio were significantly lower than that of the Control (p<0.001). The ratio was also significantly lower (p<0.01) than that of the B0 group.

In the B100 group, muscle stimulation elicited a twitch similar to Control group and significantly higher (p<0.01) than that of the B0 and B50 groups. The tetanus threshold was only different than that of the B0 (p<0.05) group. The FI was significantly lower (p<0.05) than that of the Control and B0 groups. Finally, the left muscle weight was significantly higher (p<0.001) than that of the B0 and B50 groups. However, the muscle weight/body weight ratio was always significantly lower (p<0.001) than that of the Control group but became higher (p<0.001) than that of the B50 group indicating a slight muscle mass recovery.

Numerical data concerning these parameters are presented in Table 1.

**Table 1:** Gastrocnemius muscle properties.

		Control group	BoNT/A injected groups			Comparison
			B0 group	B50 group	B100 group	
**Muscle Stimulation**	Twitch Amplitude (mN)	222.34±8.94	92.17±16.01	125.20±27.08	203.80±5.97	p<0.001
			***	**	##	
					$ $	
**Tetanus Contraction**	Tetanus Threshold (Hz)	33.75±2.26	24.95±2.29	29.29±1.70	36.25±1.25	p<0.001
(5 Hz stepwise increase frequency)			*		#	
**Electrically Induced Fatigue (10 Hz)**	Fatigue Index (Δ%)	75.92±5.47	80.07±7.37	43.07±1.87	38.64±7.78	p<0.001
				**	**	
				###	##	
**Muscle Properties**	Muscle Weight (g)	2.25±0.04	1.07±0.04	1.07±0.07	2.09±0.17	p<0.001
			***	***	###	
					$ $ $	
	Muscle Weight/ Body Weight ratio	0.48±0.01	0.25±0.02	0.16±0.01	0.26±0.02	p<0.001
			***	***	***	
				##	$ $ $	

Comparison were performed versus Control (*, p<0.05; **, p<0.01; ***, p<0.001), B0 (##, p<0.01; ###, p<0.001) or B50 ($, p<0.05; $ $ $, p<0.001) group.

### 3. Metabosensitive afferent responses

Muscle afferent fibers identified as metabosensitive fibers have spontaneous tonic low frequency baseline activity (4–10 Hz) under our experimental conditions. After potassium chloride (KCl) or lactic acid (LA) injections, or electrically-induced fatigue (EIF) an increase of the baseline tonic activity was observed.

#### Responses to electrically-induced fatigue (EIF)(S1 File)

**Fig 1:**
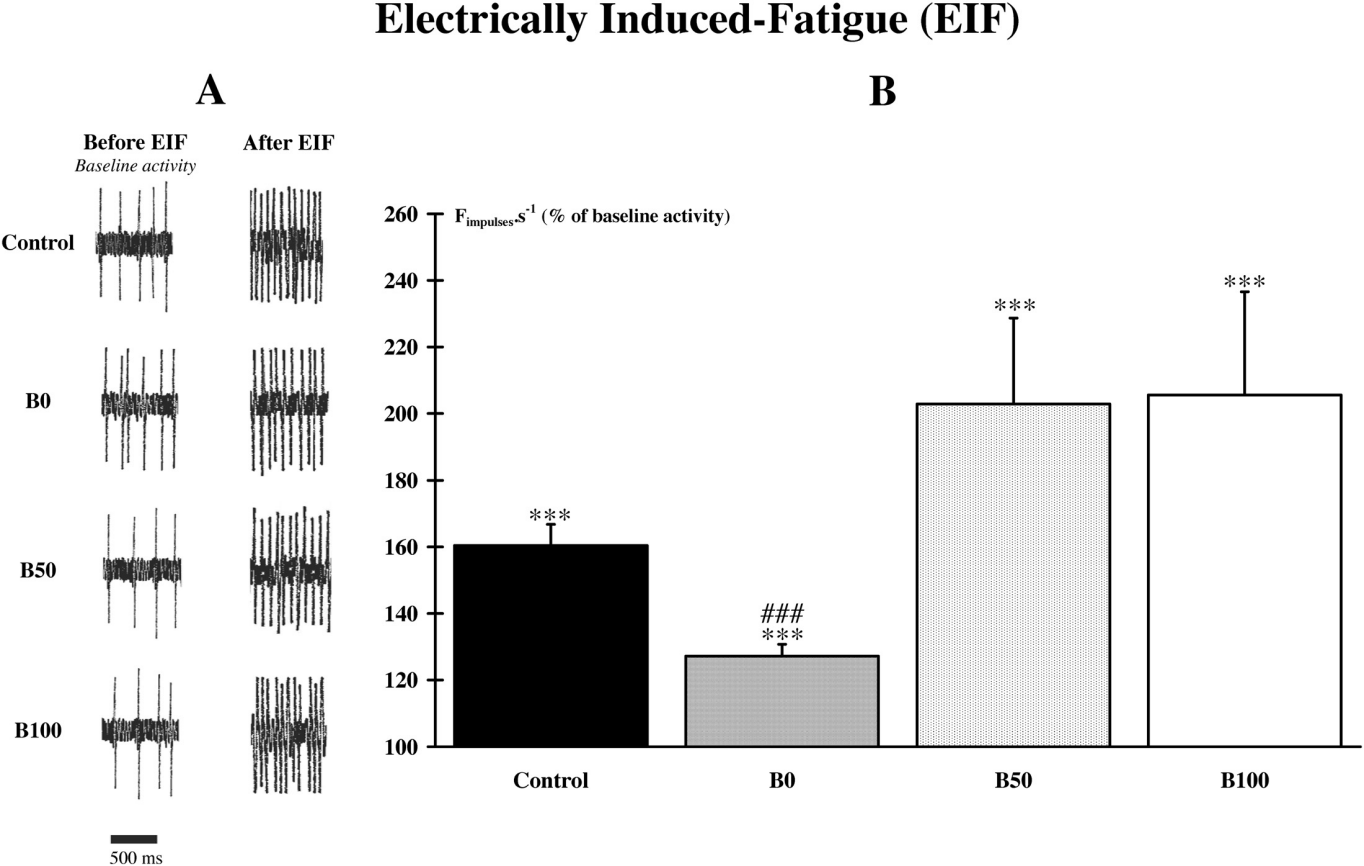
Response of the metabosensitive fibers to electrically induced fatigue (EIF). Animals from all groups exhibited a significant (***, p<0.001) increase in afferent discharge frequency after a 3-min stimulation of the *gastrocnemius* muscle. **A.** Examples of recordings before (baseline activity) and after EIF. **B.** Comparison between the post-EIF changes indicate that the mean discharge rate was significantly (###, p<0.001) lower in the B0 group compared to the 3 other groups.

The metabolites release by the repetitive stimulation in the muscle interstitium activated muscle free afferent endings. Thus, animals from all groups exhibited a significant (p<0.001) increase in afferent discharge frequency after a 3-min stimulation of the *gastrocnemius* muscle. The activation of muscle afferents was immediate when the stimulation stopped, and persisted during 3 min with a maximal response in the 2 first minutes. Intergroup comparison indicated that there is a significant (p<0.001) difference between groups. Data indicate that the mean discharge rate was significantly (p<0.001) lower in B0 group compared to the 3 other groups (Fig 1).

#### Responses to chemical injections (S2 File)

**Fig 2:**
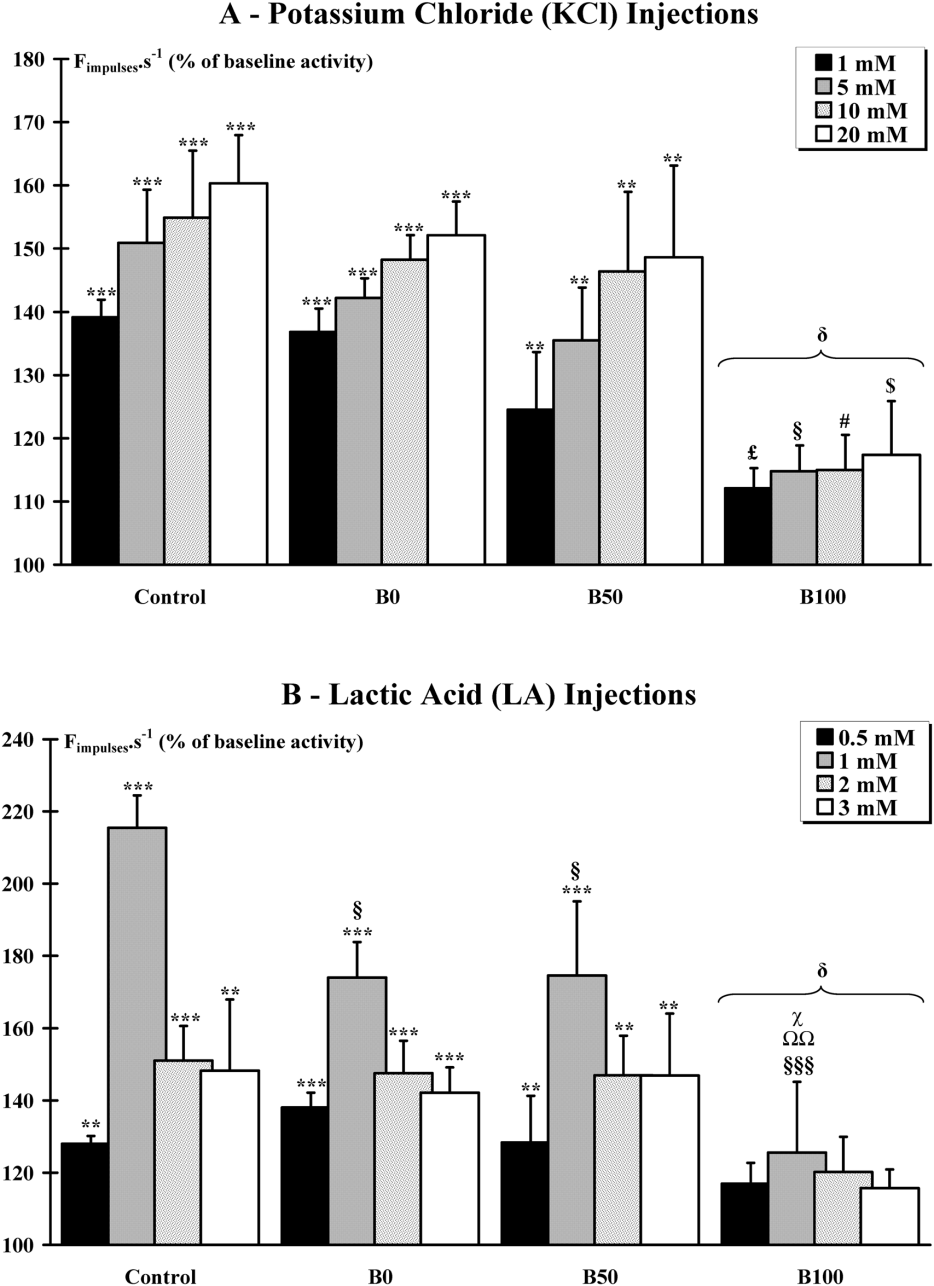
Response of the metabosensitive fibers to chemicals. Responses (F_impulses_.s^-1^) of tonically active muscle afferents during stepwise increase in potassium chloride (**A**) and lactic acid (**B**) concentration in injected solutions were recorded. Significant (**, p<0.01 and ***, p<0.001) increases in afferent discharge frequency, as compared to baseline recording, were observed in the Control and BoNT/A injected animals from B0 and B50 groups at all concentrations of KCl or LA solution tested. **A.** Intergroup comparison indicated that there is a significant difference (δ, p<0.05) in the KCl curve dose response for B100 group compared to other groups. Responses to KCl were significantly decreased in the B100 group for all concentrations. Compared to the respective concentrations of the other groups, the responses were significantly lower for 1 mM (£, p<0.05), 5 mM (§, p<0.05), 10 mM (#, p<0.05) and 20 mM ($, p<0.05). **B.** Intergroup comparison indicated that there is a significant difference (δ, p<0.05) in the LA curve dose response for B100 group compared to other groups. Responses to LA were significantly decreased for the 1 mM concentration in all BoNT/A groups (B0: §, p<0.05; B50: §, p<0.05 and B100: §§§, p<0.001) compared to the respective concentration of the Control group. Moreover, at the concentration of 1 mM, the B100 group also exhibited significant differences with B0 (ΩΩ, p<0.01) and B50 (χ, p<0.05) groups.

The pattern of responses of metabosensible afferent fibers from groups III and IV to chemical stimuli consisted of a burst of discharge beginning within 5–10 s after the bolus injection and a return to baseline values within the 3 next minutes. In a previous experiment performed in the *Tibialis anterior* muscle of Sprague-Dawley rats, we showed there was a relationship between the doses of KCl and the change in afferent discharge rate, whereas the activation of muscle afferents by LA culminated for the 1 mM concentration, and then declined [27]. Here, we showed that the *gastrocnemius* afferent fibers patterns of response were similar when KCl (Fig 2A) and LA (Fig 2B) were injected in the blood vessel irrigating the muscle. Significant (p<0.01 and p<0.001) increases in afferent discharge frequency, as compared to baseline recording, were observed in the Control and BoNT/A injected animals from B0 and B50 groups at all concentrations of KCl or LA solution tested.

Intergroup comparison indicated that there is a significant difference (p<0.05) in the KCl curve dose response between groups. In the B100 group, KCl injections did not induce a significant discharge rate increase compared to baseline. Compared to the respective concentrations of the other groups, the responses were significantly (p<0.05) lower for 1 mM, 5 mM, 10 mM and 20 mM.

Intergroup comparison indicated that there is a significant difference (p<0.05) in the LA curve dose response between groups. At the dose of 1 mM, the afferent response was significantly lower (p<0.05) in the B0 and B50 groups compared to Control group. Furthermore, the B100 group exhibited a lowered response for the 1 mM compared to the Control (p<0.001), B0 (p<0.01) and B50 (p<0.05) groups.

### 4. Mechanosensitive afferent responses

**Fig 3:**
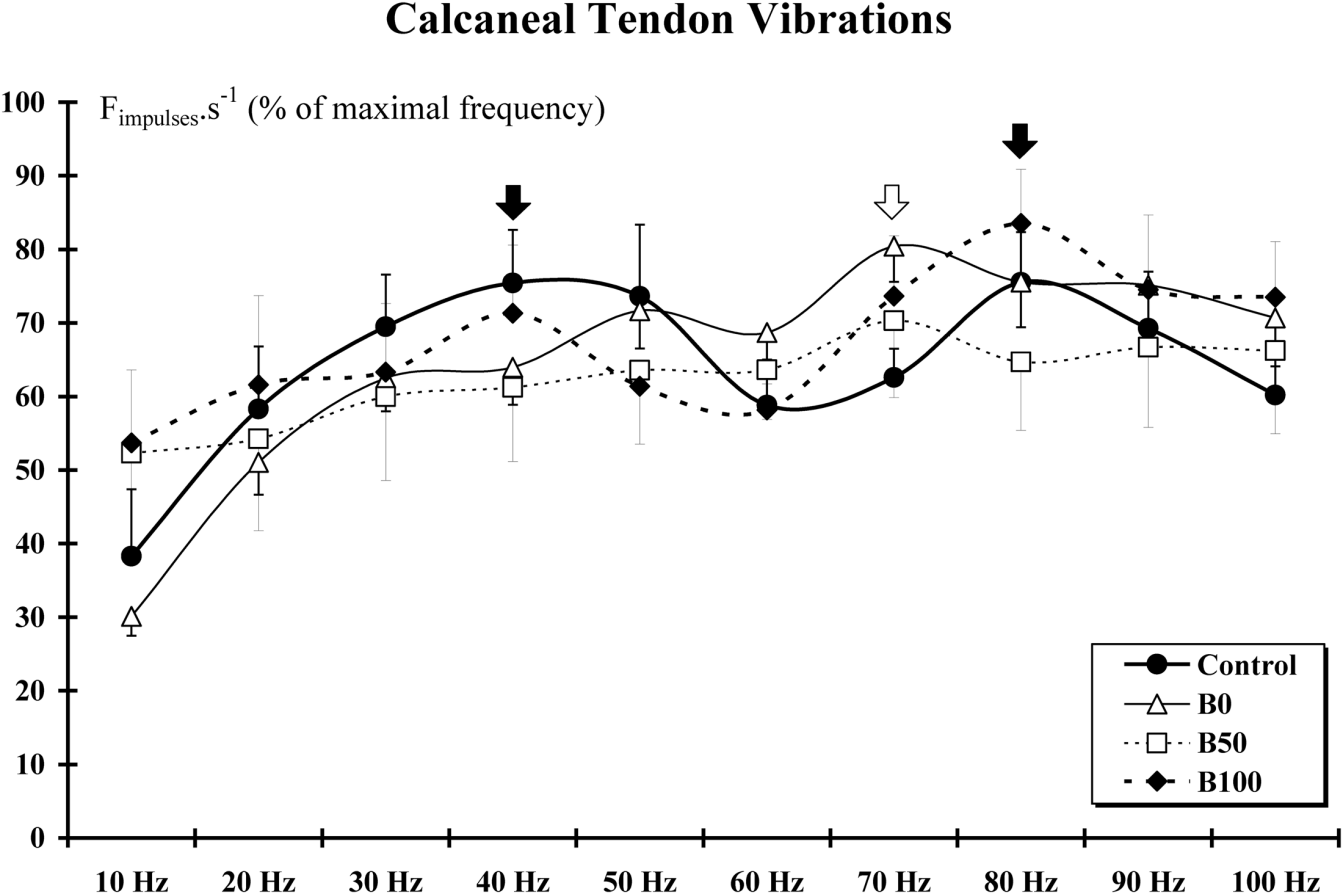
Response of the mechanosensitive fibers to calcaneal tendon vibrations. In all groups, a response (F_impulses_.s^-1^) persisting throughout the tendon vibration period was recorded for each vibration frequency. In Control and B100 groups, the changes in afferent discharge evoked by vibration are bimodal with peaks measured at 40 and 80 Hz (*black arrows*). In the B0 and B50 groups, only one peak is measured at 70 Hz (*white arrow*).

*Calcaneal* tendon vibrations resulted in an abrupt increase in the mechanosensitive afferent discharge frequency, which persisted throughout the tendon vibration period. In Control group, the rate of firing units was non-linearly related to the frequency of vibration, i.e., changes in afferent discharge evoked by vibration were bimodal with peaks measured at 40 and 80 Hz (S3 File). Thus, as previously described for the *tibialis anterior* muscle [27], two populations of muscle afferents were identified in our Control group with respect to the frequency of vibration giving an optimal activation; 66% and 34% of the units responding below and over 50 Hz, respectively. In the B0 group, these proportions were 22% and 78% while in B50 and B100 groups they were equally distributed below and above 50 Hz. When we consider the response to vibration frequencies only lower than 50 Hz, the maximal response was observed at 40 Hz (Control, B50 and B100 groups) and 50 Hz (B0 group). This maximal response was at 70 Hz (B0 and B50 groups) and 80 Hz (Control and B100 groups) for units responding maximally at frequencies higher than 50 Hz (Fig 3).

## Discussion

Previously, we demonstrated that responses of mechano- and metabosensitive afferent fibers were rapidly altered after full motor denervation, occurring within 12 days after a single injection of botulinum toxin complex type A from *Clostridium Botulinum* [19]. The experiment described in the present paper was a continuation of this previous work, and was designed to study if alterations of the mechano- and metabosensitive afferent fibers persist after toxin recovery, when locomotor function was partially and fully restored with a follow-up over many months.

Recording of afferent activities at rest and after use of specific activators indicate that neurotoxin induce long lasting changes in mechano- and metabosensitive responses despite paralysis recovery. Indeed, after half recovery (B50 group), the mechanosensitive response to tendon vibrations was still altered and the metabosensitive response was not recovered for KCl and LA injections but was exaggerated for EIF. Furthermore, in the B100 group, in which we observed a full locomotor and muscle mass recovery, the metabosensitive responses to KCl and LA were even more degraded than those of the B0 and B50 groups, and the response to EIF was still excessive. Thus, our results point out that efferent recovery is not always associated with afferent recovery.

In our study, the half and full recovery occurred at 128.43±7.43 and 371.83±24.82 days after a 15 U.kg^-1^ BoNT/A injection, respectively. This recovery time is consistent with data reported in the literature. Indeed, Sloop et al. studied the human muscle paralysis resulting from intramuscular injections of BoNT/A by measuring the *extensor digitorum brevis* M-wave amplitude after injection with 17 different doses of toxin (from 1.25 to 480 units) in healthy volunteers [45]. Two weeks post-injection, the maximal paralysis was 70 to 80% with 7.5 to 10U and at 57 weeks post-injection 22% of the original muscle paralysis was still present. In a rat model, Ma et al. reported that muscle mass, motor evoked potential and muscle force were significantly reduced during 1–2 weeks after injection of BoNT/A into the *gastrocnemius* muscle at the dose of 6 units/kg body weight but returned to nearly normal at 6 months post-injection and that the neuromuscular junction morphometry normalized at 1 year [46]. Finally, Billante et al. reported that the percentage of neuromuscular transmission was around 35% at the 200^th^ day after injection of a dose of 10U in the *gastrocnemius* muscle of rat [35]. Duration of the toxin action is mainly determined by the life-time of the toxin's light chain in the cytosol. In the present experiment, the presence of the toxin in the cytosol of terminals innervating the *gastrocnemius* muscle appears to be at least 1 year.

### 1. Muscle properties

It was previously reported that toxin recovery depend of different factors such as the rate of toxin degradation within poisoned nerve terminals, the rate of repair of the poisoned nerve terminals, the rate of new (non-poisoned) nerve terminal growth, recruitment of resting nerve terminals that escaped poisoning and a return of normal muscle mass [47]. In the B100 group, only the muscle weight/animal weight ratio remain lower than the Control group indicating that muscle mass recovery was not linearly related to the animal weight gain over time, i.e., the animal weight increasing faster than the injected muscle.

The muscle fibers of the *gastrocnemius* are mostly type II fast-twitch fibers that have an anaerobic metabolism used to create short bursts of strength and are prone to rapid fatigue [48,49]. The fall of the FI values over time should indicated a phenotype switch toward a less fast phenotype. However, the tetanus threshold and strength decreased for B50 and returned to value similar to Control when the recovery was full. These results are in accordance with previous studies showing a switch from IIb to IIa/x muscle fibers (i.e. a slight slowing of muscle contraction) associated with muscle atrophy and a reduction of force output after BoNT/A injection [50,51]. Because a higher oxidative activity for IIa/x compared to I muscle fibers was previously shown in the rat [52], this switch induces both a greater strength and fatigue resistance. BoNT/A injection impaired acetylcholine release at the neuromuscular junction leading to partial or full muscle paralysis that induces changes in contractile material and decrease in muscle cell cross sectional area. As previously suggested, changes in muscle phenotype and decrease in motor inputs (and motor units recruitment) lead to reduce muscle strength [53].

All explanations based on aging process should be excluded because the literature indicates that muscles reach their maturity by 12 months of age then decline after 18 months of age in rat [54–56].

### 2. Evoked metabosensitive activities

Group III myelinated and group IV unmyelinated afferents act primarily as mechano- and metabosensory nerve endings respectively. However, some group III fibers respond to metabolic stimuli and some group IV fibers respond to mechanical stimuli [57]. As previously demonstrated, potassium is a specific activator of metabosensitive afferent fibers and not a non-specific stimulus acting by depolarizing all fibers [27,58,59]. Furthermore, arterial KCl injections increase the potassium concentration in the muscular interstitium to levels similar to those evoked by static contractions known to activate metabosensitive afferents in rats [60], rabbits [61], cats [62] and dogs [31,63]. Lactic acid (LA) is also known to activate metabosensitive afferents via acid-sensing (proton-gated) ion channel 3 (ASIC3) and/or transient receptor potential vanilloide 1 (TRPV1) receptors [64]. Previous works [27,31] also reported that the discharge rate of metabosensitive fibers in response to increased [KCl] was concentration-dependent or culminated at 1 mM for LA. In Control and in BoNT/A injected groups, we confirmed not only this observation but also that the response of metabosensitive afferent fibers to KCl and LA injections is not (KCl) or slightly ([1 mM LA]) altered in the B0 and B50 groups. The altered response to LA injections could be explained by a reduced expression of the TRPV1 receptor into the afferent terminals which is not due to transcriptional downregulation but to the inhibition of the TRPV1 trafficking to the plasma membrane and proteasome-mediated degradation in the cytoplasm [65,66].

The unaltered responses to KCl injections in the B0 and B50 groups should be explain by the fact that ASIC3 and TRPV1 receptors are not involved in the activation of metabosensitive fibers by KCl. Unexpectedly, we observed altered responses in the B100 group with a full motor recovery after KCl and LA injections. Two explanations should be proposed. First, this mismatch between fall in metabosensible activity and increase in motor activity indicate that there is no link between the recovery of the two pathways. Second, the switch in muscle typology, as suggested above, should be responsible of this alteration, i.e., the afferents innervating a IIa/x phenotype muscle being less sensitive to KCl and LA or the discharge rate of these afferents was already near maximal at rest with no possible further activation by KCl and LA. Furthermore, we observed that EIF still induced a response in the B50 and B100 groups contrary to the B0 group. In the B0 group, the slight response of the III and IV afferent fibers should be explained by either a strong muscular atrophy altering metabolic production and then a lesser afferent activation, either by the decrease of the neurogenic inflammation (the BoNT/A impairing the substance P and CGRP release) participating to the production of inflammatory products known to activate the afferent fibers [67–69]. In the B50 and B100 groups, we can suggest that the altered metabosensitive response from group IV afferents was over compensated by group III afferents during repetitive contractions as suggested by Smith et al. [70,71] when metabosensitive afferent fibers from group IV were silent. We can conclude that the alteration observed in the B100 group is due to the group IV metabosensitive afferent fibers. This alteration could probably impact the exercise pressor reflex (EPR) adjusting the heart rate, ventilation and blood pressure during the performance of normal, daily tasks and ambulation and then indirectly the muscle mass. As previously described, skeletal muscles seem to be innervated by autonomic nervous system [72–74] and it was observed the presence of 1) adrenergic α_1_- and α_2_-receptor phenotypes expressed in higher proportion in muscles that are highly vascularized [75] or α_1_-receptor subtype in atrophied slow muscles of hypokalemic rats [76] and 2) adrenergic β_1_ and β_2_-receptor phenotypes in slow and fast-twitch muscles with an abundance of the β_2_ subtype on slow muscles [72,77–81]. Adrenergic α-receptor phenotypes seem to be implicated in vasoconstriction [82] while activation of adrenergic β-receptors induce muscle hypertrophy [83–86], vasodilatation [87] and are thought responsible for skeletal muscle apoptosis [88,89]. Furthermore, recently, it was shown that EPR mediated by III and IV afferent fibers [90] induces adrenergic activation [91]. In our experiment, because responses of III and IV afferents were changed after *gastrocnemius* BoNT/A injection, we cannot exclude an EPR down regulation in the B0 group and then a diminution of catecholamine release also contributing to muscle atrophy. In the B50 and B100 groups, the exaggerated response to EIF that could up-regulate the EPR recovery and then participate to the muscle mass recovery.

### 3. Evoked mechanosensitive activities

It is well known that muscle vibrations induce several effects on tonic and phasic reflexes [92]. Indeed, vibrations induce the muscle to slowly develop tension (tonic contraction) due to activation of anterior horn cells by the resulting afferent spindle discharge, which reaches α-motoneurons *via* monosynaptic pathways [93,94]. The literature describes that muscle spindle discharges to vibrations, and its sensibility is increased by stimulation of both types of fusimotor fibers [43]. However, mechanical vibrations were unable to activate metabosensitive afferent fibers from groups III and IV [42]. In the present study, the B0 and B50 groups presented an altered response to vibrations. This alteration was observed for frequencies of 50 and 80 Hz known to induce the greatest response in the control animals [19]. In the B0 and B50 groups, the incomplete α- and γ-motoneurons acetylcholine release induced by BoNT/A impairs the muscle spindle adjustments and then Ia and II afferents response. This decrease of influx transmission to the spinal level should, in turn, decrease the α- and γ-motoneurons discharge and so on, and then increasing the muscle paralysis. This vicious cycle, which is the opposite of the vicious cycle inducing hyperexcitabilty should delay the recovery.

Several explanations can be advanced to explain the alterations observed in the response of mechanosensitive afferents. Indeed, the literature reported that motor denervation by botulinum toxin such as with nerve injury induces a decrease in M-wave amplitude [45] and a depolarization of the resting membrane potential in the denervated muscle [95–98]. This resting membrane depolarization is associated to a reduced quantal content of subthreshold end-plate potentials and an increase in muscle fatigue [99]. The recovery is associated by a slow recovery of the quantal release and decrease of acetylcholine receptor sensitivity. More recently, Paterson et al. hypothesized about the cellular mechanisms that could be responsible for the altered mechanotransduction after BoNT/A injection [100]. Indeed, in cultured rodent primary sensory neurons, they reported a decreased in the proportion of neurons expressing slowly adapting mechanically gated currents linked to mechanical pain transduction [101]. They suggested that TRPA1 channel is required for the generation of a slowly adapting current in a subset of peptidergic dorsal root ganglion neurons [102] because its blockade reduces action potential firing in response to noxious peripheral mechanical stimulation [103] but TRPA1 channel is not sufficient alone for mechanotransduction [104]. They also suggested that there is probably no direct pharmacological action on mechanosensitive channels but an effect of BoNT-A on the trafficking of mechanosensitive channels, i.e., the tetanus toxin suppress the mechanically gated currents by blocking vesicle trafficking [105]. Vesicular trafficking plays also a role in the transmission of nerve influx at the terminals of the mechanosensitive afferent fibers [106]. Indeed, it is involved in the insertion of ion channels into the cell membrane and transport of proteins such as proteins of SNARE (soluble N-ethylmaleimide-sensitive factor attachment protein receptor) complex [107]. Thus, the alteration of the visicular trafficking by the toxin could affect the genesis of nerve impulses at the nerve endings of mechanosensitive neurons.

Finally, we observed a complete recovery in the B100 group indicating normal muscle spindle adjustments and then Ia and II afferents response, and suggesting a recovery in vesicle trafficking and muscle membrane conduction. However, although we observed a complete recovery in the B100 group at 371.83±24.82 days after BoNT/A injection we can not claim that there was no trace of toxin in the nerve endings. Following administration, only a minority of the toxin enters nerve cells. The remaining portion remains in the extracellular compartment. When the toxin enters in the terminals, it undergoes an immediate metabolic transformation. During its translocation in the cytosol, the disulfide bridge linking the heavy and light chains is broken, releasing the light chain to express its catalytic activity in the cytosol [108,109]. This process marks the end of the intracellular existence of the intact and biologically molecule. Even if the light chain or heavy chain were to be exported from the cell, only the holotoxin possesses the ability to process through the multiple steps that culminate in blockade of transmission [110]. To date, although several mechanisms of metabolism and elimination of the toxin have been hypothesized, the literature does not reported a full understanding of these mechanisms in either the extracellular or intracellular compartments [110]. A pharmacokinetic study indicated that the half-life of the toxin is about 255 min in the blood of rats [111]. However some active toxin can be detected in serum 25 days after the onset of botulism in human [110]. It seems that the persistence of BoNT intoxication can be influenced both by the ability of the toxin protease or its cleaved SNARE protein substrate to resist turnover. Finally, Shoemaker and Oyler explained the several reasons why it is not practically possible to measure the remarkable persistence BoNT/A intoxication which results from retention of active BoNT/A protease within the terminals [112].

### 4. Conclusion

In the present experiment, we showed that injection of BoNT/A in a *gastrocnemius* muscle induced an early decrease of the metabosensitive afferent response that is aggravated or exaggerated even when locomotor activity and muscle mass recovered contrary to the mechanosensive afferent response that seemed to recover over time. This pattern of response should be due to a down regulation of afferents from group IV associated with an up regulation of afferents from group III, as suggested in other pathology [71]. This change of operating mode could interfere with the functioning of the sensorimotor loop and then adjustment of muscle contractions and physiological reflexes during motor activities leading to an early fatigue. As previously described, an EPR overactivity is associated to a chronic hypertension that induces a pathological hypertrophic cardiac remodeling leading to heart failure [113]. Furthermore, the toxin transports along motor (retrograde) and sensory (anterograde) axons [114,115] could induced a direct alteration of the glutamate, substance P or GABA transmission in the central neurons [11,116,117] that could contribute further to disturb the spinal networks involved in sensorimotor activity and also to reduce pain sensation [118] and neurogenic inflammation [69].

Motor denervation with *botulinum* toxin in patients suffering of movement disorders or other pathologies, and in whom it was injected neurotoxin for cosmetic purposes, may still present proprioception and physiological alterations several months after the motor effects of the toxin have disappeared. Clinicians should keep in mind that the effects of *botulinum* toxin are sustainable and it induces alterations that are still present even after a complete motor recovery.

## Supporting Information

S1 File (XLS)

S2 File (XLS)

S3 File (XLS)
